# A well-preserved respiratory system in a Silurian ostracod

**DOI:** 10.1098/rsbl.2018.0464

**Published:** 2018-11-07

**Authors:** David J. Siveter, Derek E. G. Briggs, Derek J. Siveter, Mark D. Sutton

**Affiliations:** 1 School of Geography, Geology and the Environment, University of Leicester, Leicester LE1 7RH, UK; 2 Department of Geology and Geophysics, and Yale Peabody Museum of Natural History, Yale University, PO Box 208109, New Haven, CT 06520-8109, USA; 3 Earth Collections, University Museum of Natural History, Oxford OX1 3PW, UK; 4 Department of Earth Sciences, University of Oxford, South Parks Road, Oxford OX1 3AN, UK; 5 Department of Earth Sciences and Engineering, Imperial College London, London SW7 2BP, UK

**Keywords:** Herefordshire Lagerstätte, Ostracoda, respiratory system, Silurian

## Abstract

Ostracod crustaceans are diverse and ubiquitous in aqueous environments today but relatively few known species have gills. Ostracods are the most abundant fossil arthropods but examples of soft-part preservation, especially of gills, are exceptionally rare. A new ostracod, *Spiricopia aurita* (Myodocopa), from the marine Silurian Herefordshire Lagerstätte (430 Mya), UK, preserves appendages, lateral eyes and gills. The respiratory system includes five pairs of gill lamellae with hypobranchial and epibranchial canals that conveyed haemolymph. A heart and associated vessels had likely evolved in ostracods by the Mid-Silurian.

## 1. Introduction

Ostracod crustaceans, originated about 500 Mya [1], are abundant as fossil shells from the Ordovician to the Holocene and have colonized all aquatic environments. Most are benthic/nektobenthic, with pelagic species (exclusively Myodocopa) known from the Silurian onwards ([2] and references therein). Of the respiratory strategies [3–6] in living species only one involves respiration by gills. Only eight ostracod species (six Myodocopa) with soft parts preserved are known from the Palaeozoic, most documented from single specimens ([7] and references therein). The Herefordshire Konservat-Lagerstätte (approx. 430 Mya), UK, has yielded unparalleled evidence of the palaeobiology and evolutionary significance of diverse Silurian invertebrates, including four ostracod species ([8] and references therein). Here, we report a new Herefordshire ostracod that reveals the ancestry of the respiratory/circulatory system within the group.

## 2. Material and methods

Herefordshire Lagerstätte fossils occur as three-dimensional calcitic in fills in calcareous concretions within volcaniclastics [9]. The fossil (OUMNH C.36063) was ground and photographed at 20 µm intervals. SPIERS software [10] was used to remove extraneous material digitally, resolve fossil–matrix ambiguities and generate a colour-coded three-dimensional virtual reconstruction for study by interactive visualization, stereo-pairs and animation.

## 3. Systematic palaeontology

The single, holotype specimen (Oxford University Museum of Natural History**:** OUMNH C.36063) is classified as Euarthropoda, Crustacea, Ostracoda, Myodocopa, Myodocopida, Cylindroleberididae, *Spiricopia aurita* gen. et sp. nov. Latin, *spiritus*, breath of life, plus *copia*, abundance, alluding to the gills; *auritus*, eared, fancied resemblance of the posterodorsal lobes. From the Wenlock Series, Herefordshire, England.

*Diagnosis*: Cylindroleberidid with an elongate, reticulate carapace with a rostrum, adductorial sulcus, posterodorsal lobe, second maxilla bearing an epipod and five pairs of gills.

**Figure 1.**
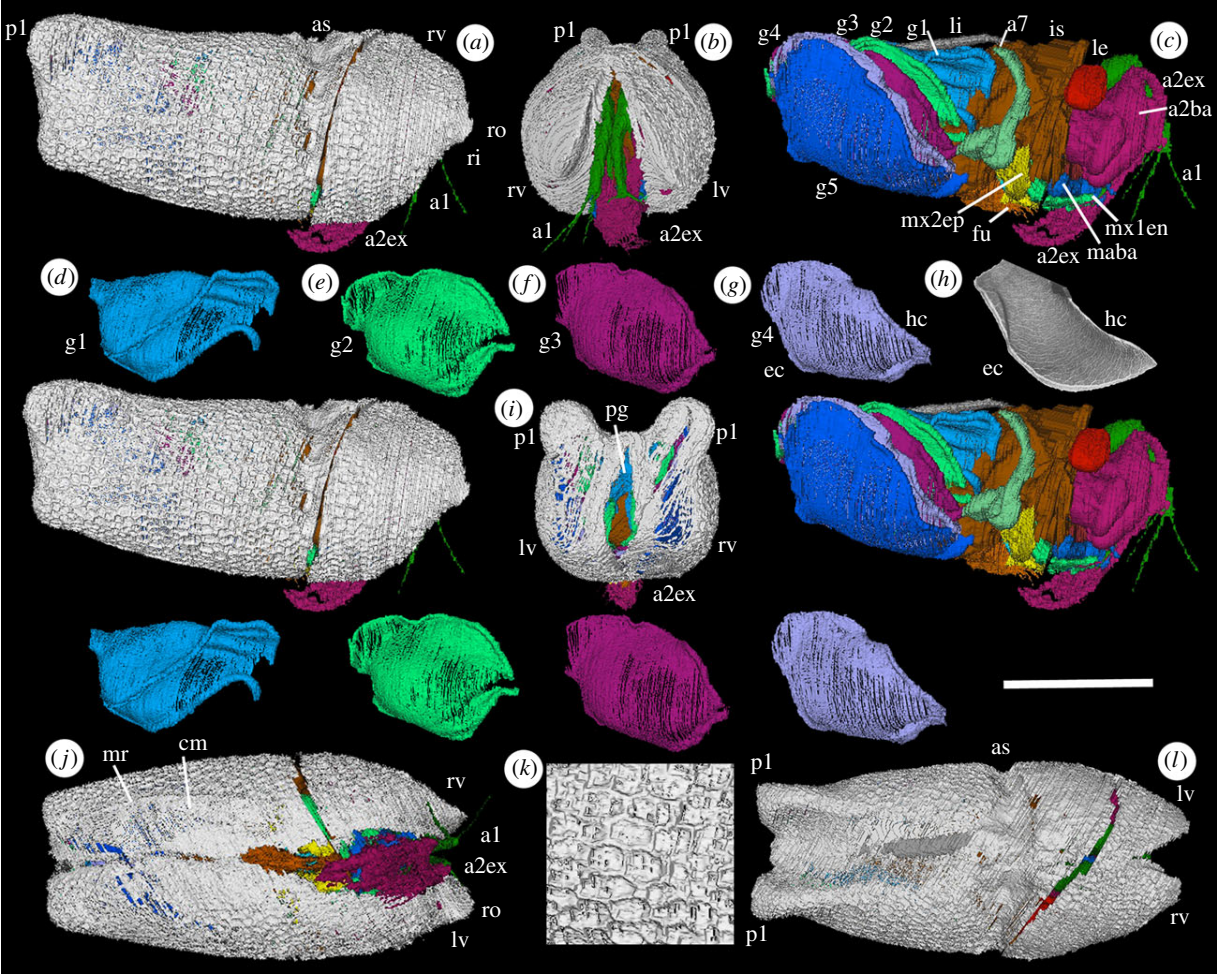
(*a–g,i–l*) *Spiricopia aurita*: ‘virtual’ reconstructions (*a,c–g*: stereo-pairs). (*a*) Right lateral view. (*b*) Anterior view. (*c*) Right lateral view, valves omitted. (*d–g*) Inner right gill lamellae. (*i*) Posterior view. (*j*) Ventral view. *(k*) Valve ornament. (*l*) Dorsal view. (*h*) Holocene cylindroleberidid *Leuroleberis surugaensis* [11], gill lamella (from [5]). Scale bar: (*a–i,l*) 2.5 mm; (*k*) 1.3 mm. a1, first antenna; a2ba, a2ex, basipod and exopod of second antenna; a7, seventh limb; as, adductorial sulcus; cm, contact margin; ec, epibranchial canal; fu, furca; g1–5, gill lamellae; hc, hypobranchial canal; is, isthmus; le, lateral eye; li, ligament; lv, left valve; maba, limb base of mandible; mr, marginal ridge; mx1en, endopod of first maxilla; mx2ep, epipod of second maxilla; pg, posterior gape; pl, posterodorsal lobe; ri, rostral incisure; ro, rostrum; rv, right valve.

*Description*: Carapace maximum length is 7500 µm; maximum height (2950 µm) and width (3200 µm) lie anterior of an adductorial sulcus (figure 1*a,l*; as). There is a beak-like rostrum above a gently concave rostral incisure (figure 1*a,j*; ro, ri). Valves gape at about 20°; a posterior gape is present (figure 1*i*; pg). The narrow adductorial sulcus occurs at one-third valve length and curves gently forward to below valve mid-height. Each valve has a prominent posterodorsal lobe (figure 1*a*,*b,i,l*; pl). A weak marginal ridge occurs in the right valve, demarcating the reticulate lateral surface from the smooth ventral (contact margin) surface (figure 1*j,k*; cm, mr); valve overlap was probably left over right.

**Figure 2.**
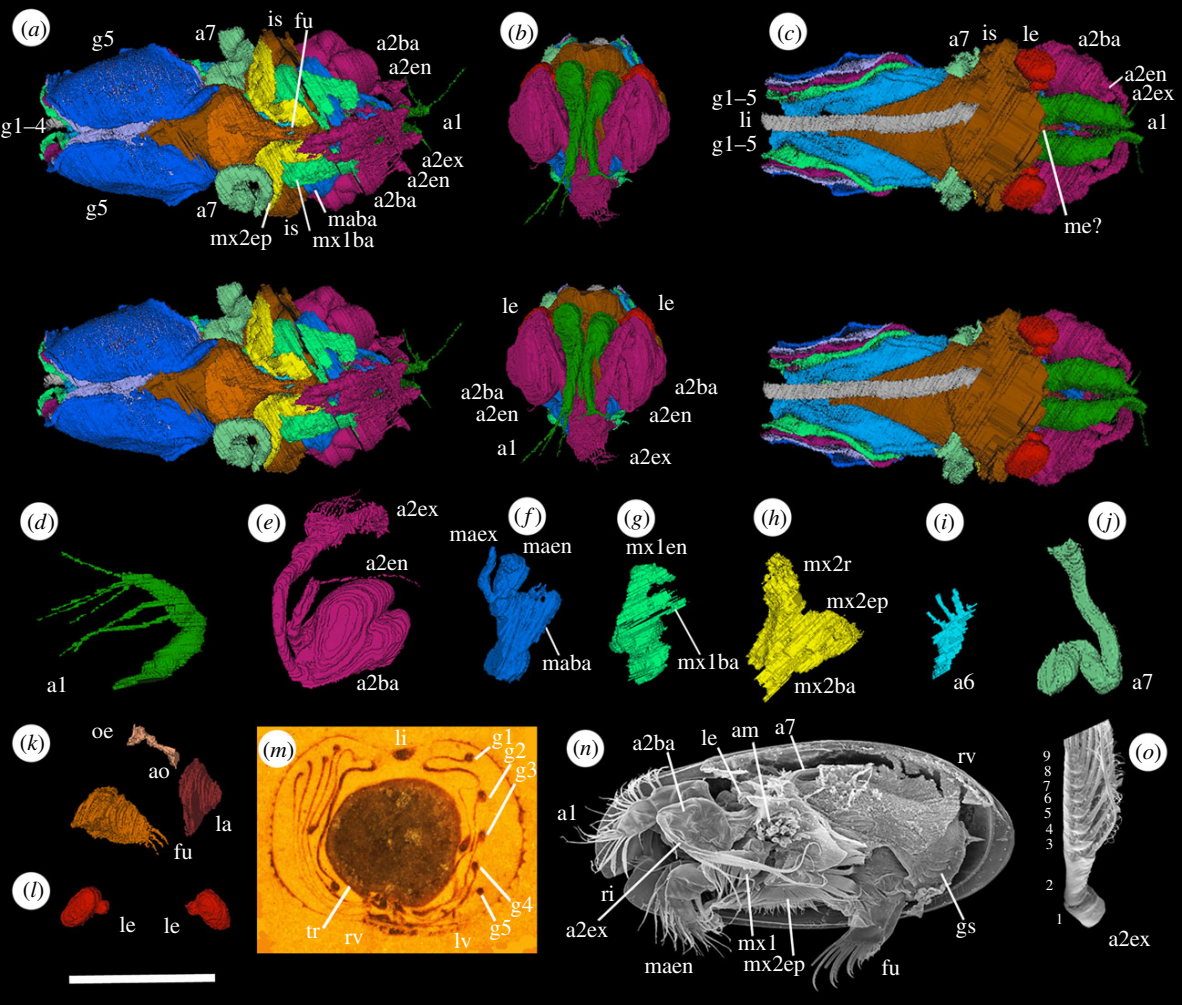
(*a–m*) *Spiricopia aurita*: ‘virtual’ reconstructions (*a–c*: stereo-pairs). (*a*) Ventral view, valves omitted. (*b*) Anterior view, valves omitted. (*c*) Dorsal view, valves omitted. (*d*) Right first antenna, adaxial face. (*e*) Right second antenna, anterior oblique view. (*f*) Right mandible, posterior oblique view of abaxial face. (*g*) Right first maxilla, adaxial face. (*h*) Left second maxilla, posterior oblique view of adaxial face. (*i*) Left sixth limb, adaxial face. (*j)* Right seventh limb, posterior view. (*k*) Oesophagus, labrum, furca, right lateral view. (*l*) Eyes, anterior view. (*m*) Specimen in rock. (*n,o*) Holocene cylindroleberidid *Asterope mariae* [12]: (*n*) internal view, juvenile (courtesy V. Perrier); (*o*) left second antenna exopod (distal part omitted; Natural History Museum, London, no. 1973:160); Scilly Isles. Scale bars: (*a–l*) 2.5 mm; (*m*) 2 mm; (*n*) 600 µm; (*o*) 300 µm. Additional abbreviations: a2en, endopod of second antenna; a6, sixth limb; am, adductor muscle; ao, atrium oris; gs, gills; la, labrum; maen, maex, endopod and exopod of mandible; me?, medial eye?; mx1, first maxilla; mx1ba, limb base of first maxilla; mx2ba, mx2r, limb base and ramus of second maxilla; oe, oesophagus; tr, trunk; 1–9, podomeres.

The first antenna (antennule: figures 1*a–c* and 2*a–d*; a1) originates close to the sagittal plane. It has a long, stout proximal part (presumed podomere) geniculate with a shorter stout middle part (presumed podomere) and a transversely compressed, longer, weakly curved distal section (podomeres not evident). Two slender setae project ventrally, at the junction of the proximal and middle podomeres and from the distal section, respectively. Four other setae, preserved only in the right limb, project from the distal podomere. A pair of pedunculate lateral eyes originate posterodorsally to the first antenna (figures 1*c* and 2*b,c,l*; le). A tiny projection between the lateral eyes may represent a medial eye (figure 2*c*; me?).

The second antenna (antenna: figures 1*a–c* and 2*a–c,e*; a2) arises laterally to the first. The basipod is large, almond-shaped, globose; a lateral depression demarcates an anterior and a smaller posterior part. The exopod is stout, strongly curved to the posterior and projects beyond the carapace. An array of long, closely spaced curved setae in the distal half of the ramus (more evident in the right limb) may reflect the podomere distribution in living cylindroleberidids (in which podomere 1 lacks setae; podomeres 2–8 each bear one seta and podomere 9 has multiple setae; figure 2*o*). The endopod is stout and a quarter as long as the exopod. It is strongly geniculate proximally, at a presumed podomere boundary. The longer distal part terminates in a long, straight seta and a shorter tightly reflexed seta.

The mandible (figures 1*c* and 2*a,f*; ma) has a broad flat limb base (presumed basipod and coxa) positioned over a small conical labrum and the presumed site of the atrium oris. Its inner edge, although poorly preserved, is serrated, suggesting enditic processes. Only a stout, proximal part (possible podomere) of the endopod is preserved. The exopod is slender, geniculate at about mid-length (possible podomere boundary) and slightly longer than the preserved part of the endopod.

The limb base (presumed basipod and proximal endite) of the first maxilla (maxillula: figures 1*c* and 2*a,g*; mx1) is broad, flat and projects adaxially over the presumed site of the atrium oris; its inner edge is poorly preserved. The ramus (presumed endopod) is large, blade-like, and consists of subequal proximal and distal parts (possible podomeres) joined at a marked geniculation. An exopod is not discernible.

The second maxilla (fifth limb: figures 1*c* and 2*a,h*; mx2) is poorly preserved. The limb base bears a stout ramus (conventionally the exopod in myodocopids; but see [13]) and a large laterally projecting lamellar epipod with a curved ventral margin. A small sixth limb lies against the inner base of the fifth appendage; it consists of a short, flat ramus bearing a row of three short setae (figure 2*i*; a6). A seventh limb arises below mid-height anterior of the gills. It is vermiform, about 3500 µm long with a v-shaped termination (figures 1*c* and 2*a,c,j*; a7).

Five pairs of thin, overlapping gill lamellae flank the trunk (figures 1*c*–*g* and 2*a,c,m*; g1–5). A narrow subcylindrical swelling follows the margin of each lamella. Lamella 1 is folded, forming an outer drape posterodorsally (figures 1*c*,*d* and 2*c*; g1). Lamellae 4–5 are elongate, paddle-like, with a gently curved ventral margin.

The isthmus occurs at the adductorial sulcus (figures 1*c* and 2*a,c*; is). A non-mineralized dorsal band of soft tissue is interpreted as a ligament (figures 1*c* and 2*c,m*; li). Part of the oesophagus is preserved as a narrow sediment infill, projecting posterodorsally from behind the labrum (figure 2*k*; oe). A furca is preserved between the posterior limbs; each furcal lamella bears at least six long, slender, gently curved claws only four of which are completely preserved (figures 1*c* and 2*a,k*; fu).

## 4. Discussion

A vermiform seventh appendage and lateral eyes occur only in myodocopids, and gills are known only in Cylindroleberididae (figure 2*n*). The morphology of appendages 1–6, including the presence of an epipod only on the second maxilla, is also compatible with a cylindroleberidid placement. Many of the appendages resemble those of the Herefordshire Lagerstätte cylindroleberidids *Colymbosathon*, *Nasunaris* and *Pauline* and the associated nymphatelinid myodocopid *Nymphatelina* [14–18]. *Spiricopia* is unusual among cylindroleberidids in having a prominent adductorial sulcus and atypical in apparently lacking a setate comb on the second maxilla. The shell morphology of these five Herefordshire myodocopids is very variable and emphasizes that the carapace alone is an unreliable basis for classifying fossil ostracods [17].

Living cylindroleberidids use the setate comb for filter feeding. As a setate comb is not evident in *Spiricopia*, it likely used its furca and appendages for predation, scavenging and detritivory on or near the substrate, as do most living myodocopids [19]. Its powerful antennae suggest effective swimming.

In small ostracods (less than 3 mm long), gas diffusion occurs via the surface of the body integument and the uncalcified inner lamella integument. In larger ostracods (myodocopid and halocyprid Myodocopa) simple diffusion for gaseous exchange is supplemented by internal fluid convection via an anastomosing integumental vascular network which forms part of an integrated circulatory system [3–6]. Evidence from external shell ornament suggests that certain Silurian myodocopes had an integumental vascular network [4]. Among myodocopes, cylindroleberidids augment this with gills (external integumental folds), typically seven pairs, which, *inter alia*, enhance metabolism. The only fossil ostracods previously known to have gills are *Colymbosathon ecplecticos*, which preserves six incomplete gill pairs, and *Nasunaris flata* and *Pauline avibella*, which preserve fragmentary gill lamellae [14,16,17]. Gill-like features also occur in the Triassic myodocope *Triadocypris spitsbergensis* [20]. By comparison with living cylindroleberidids [4,5] (figure 1*h*), the swollen margin of each gill lamella in *Spiricopia* can be confidently interpreted as the site of hypobranchial (concave; afferent) and epibranchial (convex; efferent) canals that conveyed haemolymph in life (figures 1*c–g* and 2*m*). The ridge-like free edges previously noted in the gills in *Colymbosathon* [14] can be similarly interpreted. As in living cylindroleberidids [5], the epipod of the second maxilla likely functioned to rhythmically ventilate the domicilium including the gills, with a water/feeding current entering anteriorly and egressing posteriorly. The gills are posterior, the probable region for preferential oxygen exchange in myodocopids and a position that facilitates grooming by the vermiform seventh limb [4,5]. The lamellae in *S. aurita* provide a total surface area of about 35 mm^2^ for gaseous exchange in an outer shelf/slope, purported 150–200 m deep environment [21]. Whether a diurnal activity rhythm featured in tissue oxygenation, as in some living cylindroleberidids, which migrate between the sea bottom and the water column [22], is unknown. The myodocopid *S. aurita* reveals a well-developed branchial system. Likely a heart and associated vascular system had evolved in ostracods by the Silurian.

## Data accessibility

The model (VAXML/STL format) of OUMNH C.36063 is at Dryad Digital Repository: http://dx.doi.org/10.5061/dryad.000bq75 [23].
